# HPLC-PDA-MS/MS Characterization of Bioactive Secondary Metabolites from *Turraea fischeri* Bark Extract and Its Antioxidant and Hepatoprotective Activities In Vivo

**DOI:** 10.3390/molecules22122089

**Published:** 2017-11-29

**Authors:** Mansour Sobeh, Mona F. Mahmoud, Omar M. Sabry, Rasha Adel, Malak Dmirieh, Assem M. El-Shazly, Michael Wink

**Affiliations:** 1 Institute of Pharmacy and Molecular Biotechnology, Heidelberg University, Im Neuenheimer Feld 364, 69120 Heidelberg, Germany; 2 Department of Pharmacology and Toxicology, Faculty of Pharmacy, Zagazig University, Zagazig 44519, Egypt; 3 Department of Pharmacognosy, College of Pharmacy, Cairo University, Cairo 11562, Egypt; 4 Department of Pharmacognosy, Faculty of Pharmacy, Zagazig University, Zagazig 44519, Egypt

**Keywords:** *Turraea fischeri*, flavonolignan, cinchonains, HPLC-PDA-ESI-MS/MS, antioxidant, hepatoprotection

## Abstract

*Turraea fischeri* is an East African traditional herb, which is widely used in traditional medicine. In this study, we profiled the secondary metabolites in the methanol extract of *T. fischeri* bark using HPLC-PDA-ESI-MS/MS, and 20 compounds were tentatively identified. Several isomers of the flavonolignan cinchonain-I and bis-dihydroxyphenylpropanoid-substituted catechin hexosides dominated the extract. Robust in vitro and in vivo antioxidant properties were observed in 1,1-diphenyl-2-picrylhydrazyl radical scavenging assay (DPPH) and ferric reducing antioxidant power (FRAP) assay, and in the model organism *Caenorhabditis elegans*. Additionally, the extract exhibited promising hepatoprotective activities in D-galactosamine (D-GaIN) treated rats. A significant reduction in the elevated levels of aspartate aminotransferase (AST), total bilirubin, gamma-glutamyltransferase (GGT), and malondialdehyde (MDA) and increase of glutathione (GSH) was observed in rats treated with the bark extract in addition to D-galactosamine when compared with rats treated with D-galactosamine alone. In conclusion, *T. fischeri* is apromising candidate for health-promoting and for pharmaceutical applications.

## 1. Introduction

Although oxygen metabolism is a central key to life, it is also associated with reactive oxygen species (ROS) production; this phenomenon is known as the “Oxygen Paradox”. A large number of diseases, including cardiovascular diseases, stroke, cancer, diabetes, Parkinson, and Alzheimer disease, are apparently linked to ROS. Also, ageing and several age-related degenerative diseases involve inflammation and deleterious tissue damage from oxidative components. This also applies to liver cirrhosis [1].

In the body, some endogenous antioxidant enzymes are able to cope with ROS, and thus can limit the damage of ROS. However, in many diseases, among them liver injury, the antioxidant abilities are diminished or severely compromised. In the case of excessive amounts of ROS, other protective mechanisms, such as external antioxidants, could be of a great value [2,3].

The genus *Turraea* L. of the Meliaceae family comprises more than 70 species in Africa, Madagascar, Socotra, and tropical Asia; *Turraea* plants grow as trees or shrubs [4]. The genus is rich in secondary metabolites, such as limonoids, sterols, and flavonoids [5]. Several species from the genus were systematically investigated for their potential as antimalarial agents, among them, *T. vogelii*, *T. robusta*, *T. nilotica*, *T*. *floribunda*, and *T*. *fischeri* [6,7,8]. Also, some African *Turraea* species are traditionally used as an aphrodisiac and for the treatment of wounds, abscesses, asthma, and against bilharzias [9].

*Turraea fischeri* Gürke is an East African traditional herb and is widely used in folk medicine against stomachache and for fertility properties [10]. However, neither phytochemical studies nor biological investigations have been carried out for this plant. Therefore, this work describes for the first time the chemical constituents of a methanol extract of *Turraea fischeri* bark using HPLC-PDA-ESI-MS/MS. The in vivo hepatoprotection against D-galactosamine (D-GaIN) in toxication in rats and antioxidant properties in the nematode *Caenorhabditis elegans*, which is a widely used model system in this context [11,12], were evaluated.

## 2. Results and Discussion

### 2.1. Phytochemicals in T. fischeri Bark

**Figure 1:**
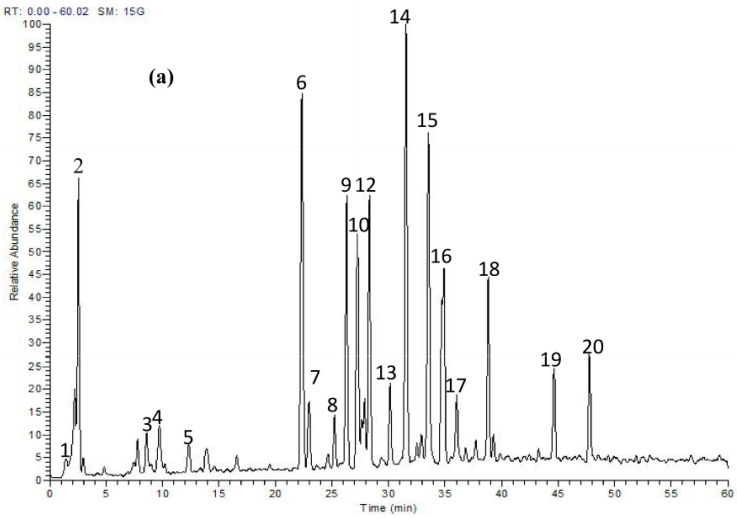

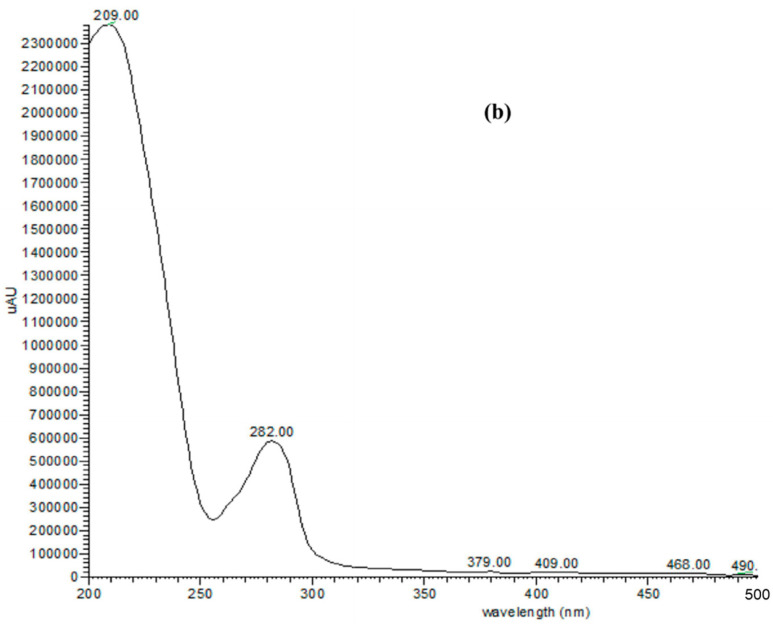
(**a**) HPLC-ESI (−)-MS/MS profile of a methanol extract of *T. fischeri* bark; (**b**) A sample spectra (UV) of compounds (**3**–**20**) at 280 nm.

**Figure 2:**
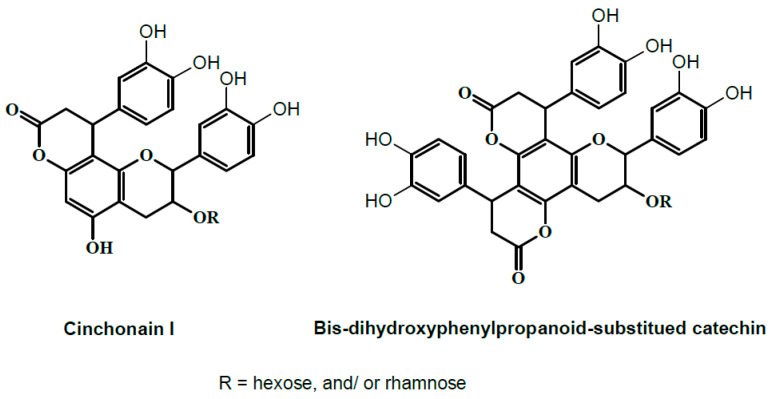
Tentative structures of some compounds from Table 1.

HPLC-PDA-MS/MS was applied to profile the secondary metabolites of a methanol extract from *T. fischeri* bark, and 20 compounds were tentatively identified (Figure 1 and Figure 2, and Table 1). Flavonolignans and bis-dihydroxyphenylpropanoid-substituted catechin isomers dominated the extract.

**Figure 3:**
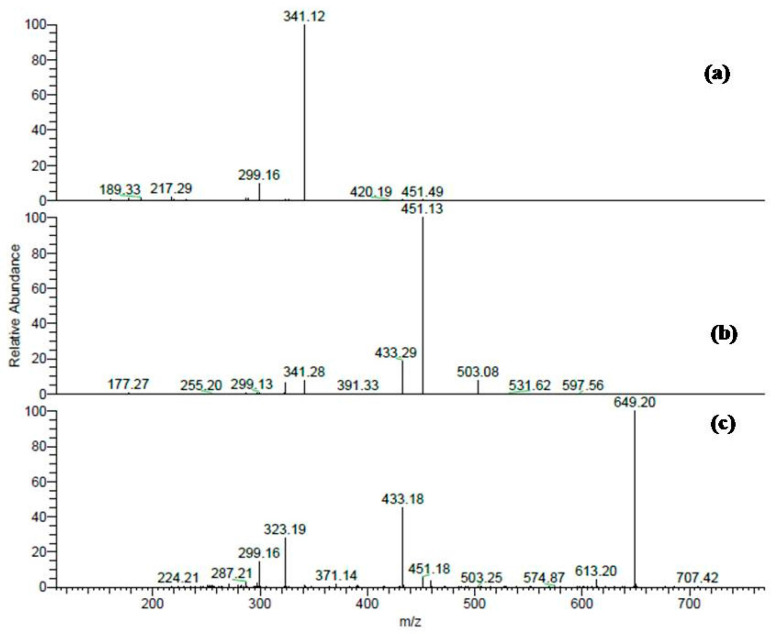
(**a**) MS/MS fragmentation of cinchonain-I at [M − H]^−^ (*m*/*z*) at 451; (**b**) MS/MS fragments of cinchonain-I hexoside at [M − H]^−^ (*m*/*z*) at 613; (**c**) MS/MS spectra of cinchonain-I rhamnosyl-hexoside at [M − H]^−^ *m*/*z* 759.

For instance, peak (**15**) at a retention time 33.41 min, exhibited a molecular ion peak [M − H]^−^ (*m*/*z*) at 451 and two daughter ions at *m*/*z* 341 and 299; it was assigned to cinchonain-I, as previously described [13] (Figure 3a). Three peaks (**8**, **9**, and **10**) at retention times 25.24, 26.27, and 27.69, respectively, showed [M − H]^−^ (*m*/*z*) at 613 and three fragment ions at 299, 341, and 451; they were identified as cinchonain-I-hexoside isomers (Figure 3b).

**Figure 4:**
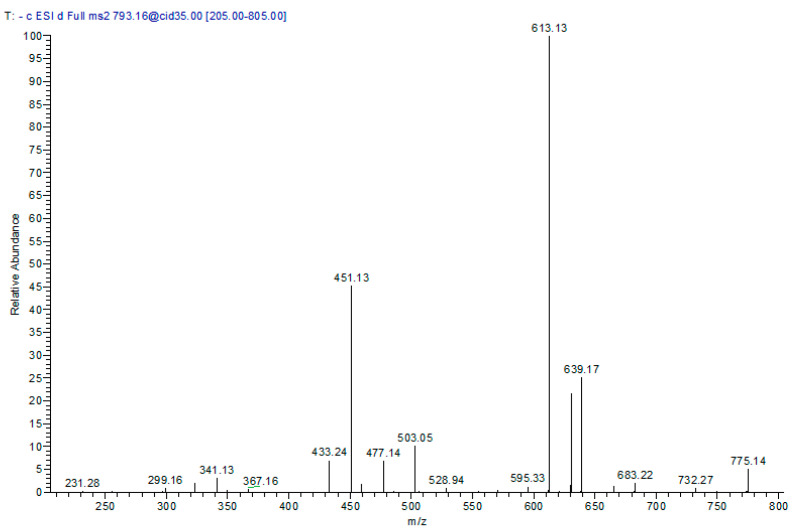
MS/MS spectra of cinchonain-I syringyl-hexoside at [M − H]^−^ *m*/*z* 793.

Also, six further compounds (**6**, **7**, **11**, **12**, **13**, and **17**) displayed similar molecular ion peaks at [M − H]^−^ (*m*/*z*) 759. In MS/MS analyses, they produced fragment ions as 649 (M–H–110), 613 (M–H–146, rhamnose moiety), and 451 (M–H–146–162, rhamnose and hexose moieties); they were characterized as cinchonain-I rhamnosyl-hexoside isomers (Table 1, Figure 3c). Additionally, a minor compound (**5**) at a retention time 12.24 min had [M − H]^−^ *m*/*z* 793 and in MS^2^ demonstrated daughter ions at *m*/*z* 613 (M–H–180, syringyl moiety) and 451 (M–H–180–162, syringyl and hexoside moieties); it was tentatively identified as cinchonain-I syringyl-hexoside (Table 1 and Figure 4).

**Figure 5:**
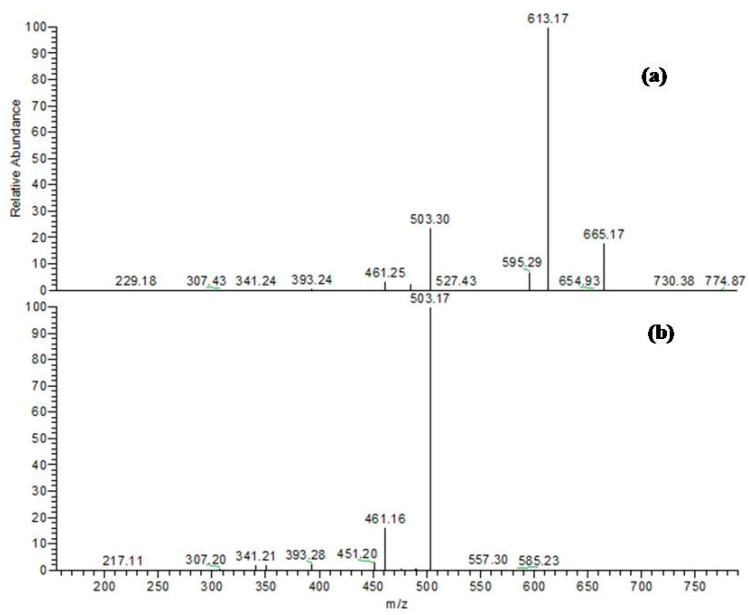
Negative ion ESI-MS/MS spectra of bis-dihydroxyphenylpropanoid-substituted catechin–hexoside; (**a**) MS^2^ of [M − H]^−^ *m*/*z* 775; (**b**) MS^3^ of main daughter ion at *m*/*z* 613.

**Figure 6:**
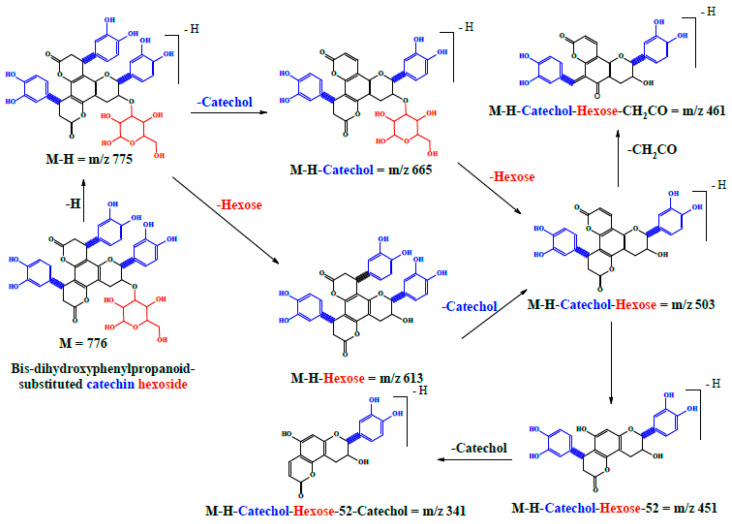
A proposed fragmentation pattern of bis-dihydroxyphenylpropanoid-substituted catechin hexoside at [M − H]^−^ *m*/*z* 775.

Two peaks at 34.76 and 47.74 min (compounds **16** and **20**) with a molecular ion of [M − H]^−^ at *m*/*z* 775 were detected. In MS^2^, they produced a major ion peak at *m*/*z* 613 [M–H–162, loss of a hexose moiety]. In MS^3^ of 613, two daughter ions at *m*/*z* 503 and 461 were observed; thus, they were identified as bis-dihydroxyphenylpropanoid-substituted catechin hexoside isomers, as reported before [13] (Figure 5a,b and Figure 6).

**Figure 7:**
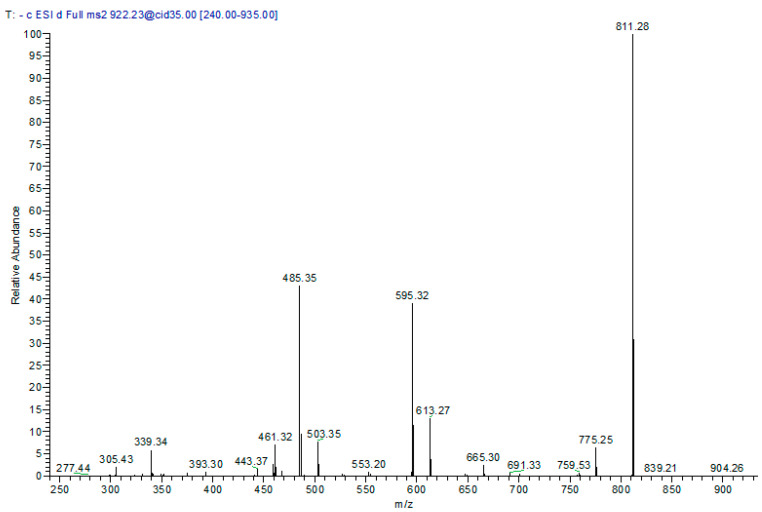
Negative ion ESI-MS/MS spectra of bis-dihydroxyphenylpropanoid-substituted catechin rhamnosyl-hexoside at [M − H]^−^ *m*/*z* 921.

In the extract, three compounds (**14**, **18**, and **19**) had similar [M − H]^−^ at *m*/*z* 921, as well as similar fragmentation pattern. In MS^2^, they produced a major daughter ion at *m*/*z* 811 due to the loss of dihydroxyphenyl group. Also, the two ions at *m*/*z* 775 and 613 were produced due to the losses of 146 and 162, respectively. Therefore, these three precursors were tentatively assigned to bis-dihydroxyphenylpropanoid-substituted catechin rhamnosyl–hexoside isomers. Their retention times are documented in Table 1 and a representative mass spectrum is presented in Figure 7. Other two minor peaks (**3** and **4**) produced a molecular ion peak at [M − H]^−^ *m*/*z* 939 and fragment ions at 759 and 613; they were tentatively characterized as bis-dihydroxyphenylpropanoid-substituted catechin syringyl-rhamnoside isomers (Table 1).

### 2.2. Antioxidant and Hepatoprotective Activities

In initial analyses, we determined the total phenolic content of a bark extract using the Folin-Ciocalteu method. The extract contained 488 mg gallic acid equivalent/g extract. Next, we assessed the in vitro antioxidant properties of this extract by DPPH (1,1-diphenyl-2-picrylhydrazyl radical scavenging assay) and FRAP (Ferric reducing antioxidant power) assay. A pronounced antioxidant activity was detected in both of the assays in comparison to the potent antioxidant compound ascorbic acid (Table 2). Comparable antioxidant properties were reported in *Trichilia catigua* and *Parabarium huaitingii* [14,15].

**Figure 8:**
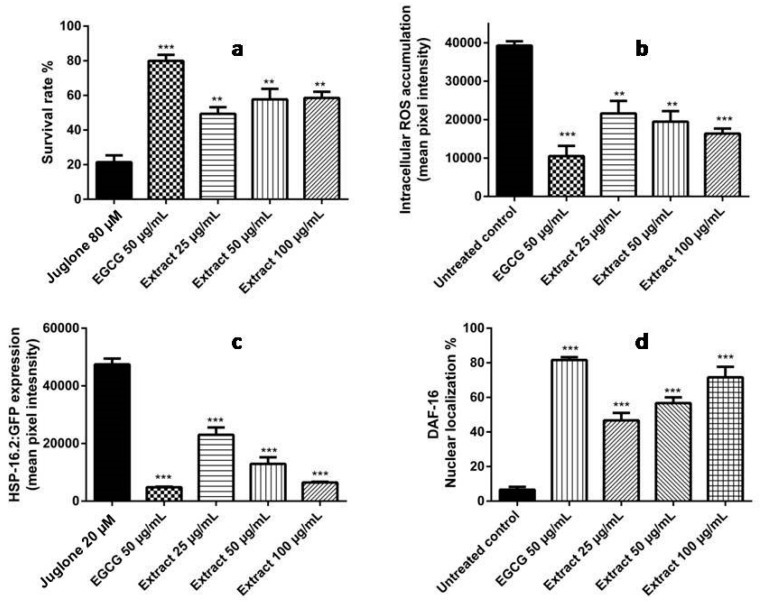
(**a**) Influence of *T. fischeri* extract on the survival rate in N2 worms against the deleterious effects of juglone (80 µM). The extract improved the survival rate to 49.39%, 57.73% and 58.54% at concentrations of 25, 50, and 100 µg/mL, respectively, when compared to the juglone-alone control (21.49%), (mean ± SEM, *n* = 3); (**b**) Influence on intracellular ROS accumulation in N2 nematodes evidenced by H2DCF-DA dye. A significant reduction was observed in ROS levels by 44.96%, 50.44% and 58.32% when the worms were treated with 25, 50, and 100 µg/mL extract, respectively; the control was set 100%. ROS levels were measured by fluorescence microscopy. Data are expressed as relative fluorescent intensity compared to control group (mean ± SEM, *n* = 3); (**c**) Influence of the extract on *Phsp*-16.2::GFP expression in mutant strains TJ375. *Phsp-*16.2::GFP levels were significantly decreased by 51.49%, 72.73%, 86.36% after pre-treatment of the nematodes with 25, 50, and 100 μg/mL extract followed by 20 µM juglone; (**d**) Influence of the extract on the localisation of the transcription factor DAF-16 in mutant TJ356 strains. The extract induced nuclear localization to 46.67%, 56.67%, and 71.67% at concentrations of 25, 50, and 100 µg/mL extract, respectively. DAF-16::GFP localization was determined using fluorescence microscopy. The worms were assigned into three groups: cytosolic, intermediate, and nuclear according to their phenotype. ** *p* < 0.01, *** *p* < 0.001 related to control was analysed by one-way ANOVA. Interestingly, the extract showed comparable activities with the reference compound epigallocatechin gallate (EGCG) in all assays.

In the next step, we investigated whether these in vitro antioxidant activities would induce protective effects in vivo in *C. elegans*. We initially studied the survival rate of the wild type N2 worms that were treated with the toxic and pro-oxidant juglone (80 µM) to generate oxidative stress that is lethal within 24 h. The pre-treatment of the worms with the extract resulted in an increased survival rate in a dose dependent manner (Figure 8a).

Given these initial findings, we went further to assess the abilities of the extract to diminish ROS production or at least scavenge it. Thus, the intracellular ROS level was quantified in N2 worms against elevated ROS levels. The extract reduced the ROS level in a concentration dependent pattern (Figure 8b).

Prolonged exposure to extreme stress conditions can harm cells, damage proteins, and thus result in cell and tissue death. Heat-shock proteins (HSPs) can be detected in all of the studied organisms, and they are increasingly expressed in the stressed cells to protect and/or repair such damage by preventing protein aggregation and misfolding, and to maintain homeostasis [16]. To explain the possible mechanism of the observed in vivo antioxidant activities of the extract, we incubated the transgenic strains TJ375 with 20 μM juglone for 24 h to induce oxidative stress, and then we measured the expression of the green fluorescent protein (GFP) in *Phsp*-16.2::GFP in the pharynx. The extract significantly reduced HSP-16.2 expression in a dose-dependent fashion (Figure 8c).

To further elucidate the mechanisms by which the extract exerts its antioxidant activities, we assessed its effects on the localization of the FOXO transcription factor DAF-16. The translocation of DAF-16 from the cytosol into the nucleus is a primary prerequisite for its ability to promote the expression of several defense enzymes, among them, superoxide dismutase and heat-shock proteins [17]. Apparently, the extract increased nuclear localization of DAF-16::GFP in daf-16-jfp reporter strain (transgenic strain TJ356), indicating a reduced DAF-16 phosphorylation status after extract treatment in a dose-dependent pattern (Figure 8d). This suggests that the in vivo antioxidant activities of *T. fischeri* extract might be mediated by the DAF-16/FOXO pathway. In conclusion, the polyphenolics in the bark extract are bioavailable and are partly taken up by *C. elegans*. The results agree with those of earlier experiments in our laboratory [18,19,20,21].

**Figure 9:**
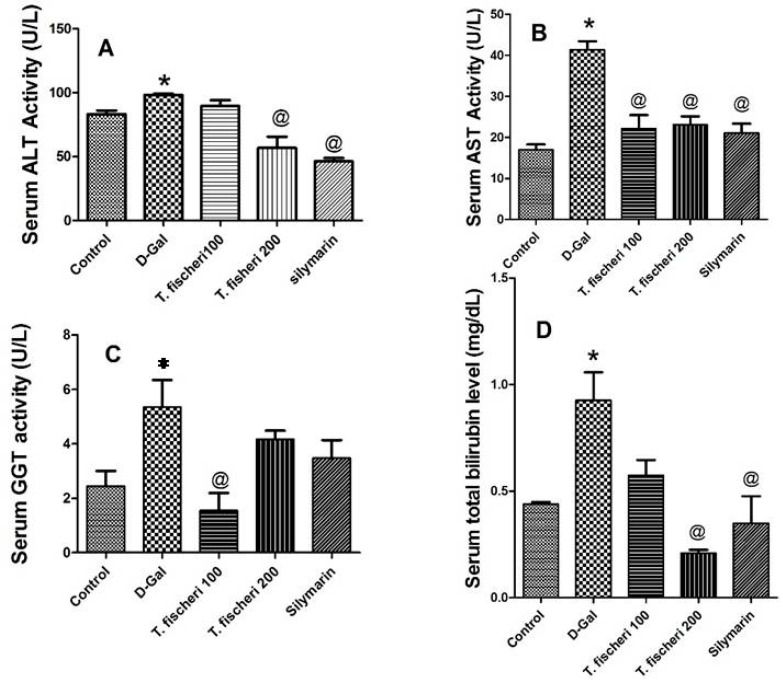
Influence of a single oral dose of d-galactosamine—induced liver injury (800 mg/kg) and oral administration of two doses (100 mg/kg and 200 mg/kg b.w.) of *T. fischeri* extract and the positive control silymarin (100 mg/kg) on serum enzyme activities (**A**) alanine aminotransferase (ALT); (**B**) aspartate aminotransferase (AST); (**C**) gamma-glutamyltransferase (GGT); (**D**) Total bilirubin level. Results are expressed as mean ± SEM. * Significant difference compared to normal control group; ^@^ Significant difference compared to D-GalN treated group at *p* < 0.05. *n* = 6; by One Way ANOVA and Tukey post hoc test.

In light of these in vitro and in vivo findings, we investigated the hepatoprotective potential of the bark extract against the severe effects of liver intoxication in the well established rat model after injection of D-galactosamine (D-GaIN) [22]. We first investigated the biochemical changes in the liver enzymes after 24 h of D-GalN injection. A significant elevation in liver enzyme activities (aspartate aminotransferase (AST), alanine aminotransferase (ALT)) and total bilirubin were observed in D-GalN-rats. Pretreatment of the rats with 200 mg/kg extract and silymarin attenuated the increase in ALT and AST activities, and total bilirubin (*p* < 0.05, Figure 8). However, pretreatment with 100 mg/kg b.w. extract significantly decreased AST and gamma-glutamyltransferase (GGT) levels when compared with D-GalN (*p* < 0.05), but did not affect ALT and total bilirubin when compared with D-GalN (*p* > 0.05, Figure 9).

**Figure 10:**
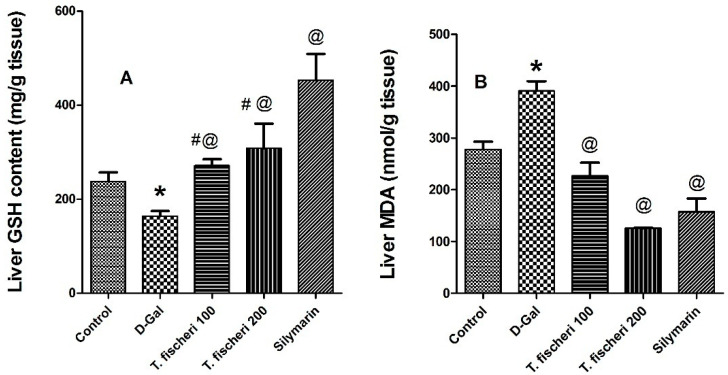
Influence of a single oral dose of D-galactosamine-induced liver injury (800 mg/kg) and oral administration of two doses (100 mg/kg and 200 mg/kg b.w.) of *T. fischeri* extract and the positive control silymarin (100 mg/kg) on (**A**) Reduced glutathione content (GSH, mg/g liver tissue); (**B**) Malondialdehyde content (MDA, nmol/g liver tissue). Results are expressed as mean ± SEM. * Significant difference compared to normal control group; ^@^ Significant difference when compared to D-GalN treated group at *p* < 0.05; ^#^ significant difference compared to silymarin treated group at *p* < 0.05, *n* = 6; by One Way ANOVA and Tukey post hoc test.

Furthermore, we studied the influence of *T. fischeri* extract on oxidative stress markers. D-GalN injection increased the generation of ROS and reduced the level of endogenous antioxidants. This resulted in an increased oxidative stress, as revealed by elevation of malondialdehyde (MDA), a marker of lipid peroxidation and depletion of glutathione (GSH) (*p* < 0.05, Figure 10). Oral administration of *T. fischeri* extract in doses of 100 mg/kg and 200 mg/kg b.w. and silymarin revealed potent antioxidant effect. It diminished the elevated MDA content and increased endogenous GSH when compared to D-GalN alone (*p* < 0.05, Figure 10). The effect of the extract in either dose levels was similar to silymarin on MDA but the latter exerted a stronger effect on GSH.

**Figure 11:**
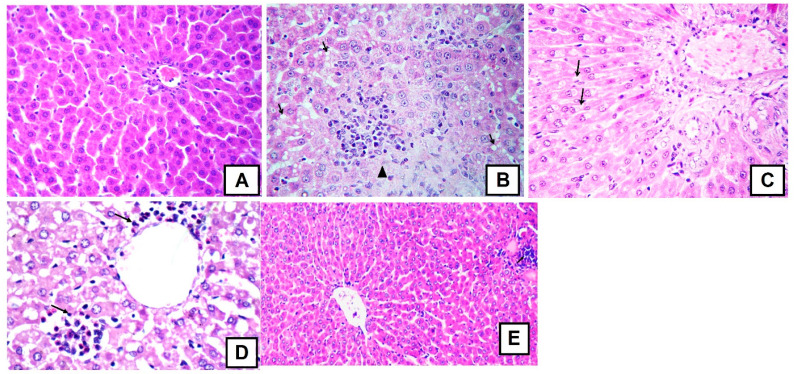
Representative photomicrographs of cross sections from six rat livers (staining with hematoxylin and eosin, 400×); (**A**) Liver of healthy rats with normal hepatocytes arranged in hepatic cords radiating from the central vein with normal portal area, central vein and normal ducts; (**B**) Liver of D-galactosamine treated rats showing focal hepatic necrosis infiltrated by mononuclear cells (arrow head) with microsteatosis of the adjacent hepatocytes (arrows); (**C**) Liver of extract (100 mg/kg) treated rats with minute fibrous strands in portal area and interlobular tissue with reversible degenerative changes mainly vacuolar degeneration in the hepatic cells (arrows); (**D**) Liver of extract (200 mg/kg) treated rats showing portal inflammation (arrows) but no steatosisornecrosis; and, (**E**) Liver of silymarin extract (100 mg/kg) treated rats showing partial improvement but some monocellular infiltration. Photomicrographs from control, D-galactosamine and silymarin groups were published before in [23], where the experiments were carried out in parallel.

Apparently, the studied extract exerted pronounced hepatoprotective activities, as evidenced by the biochemical activities of the liver enzymes and the ROS markers GSH and MDA. Additional evidence was found when the histopathological changes were investigated. D-GalN caused focal hepatic necrosis and hepatic inflammation as revealed by mononuclear cell infiltration (Figure 11B). Furthermore, acute cell swelling and intense microsteatosis were also observed in the hepatic cells. Single or multiple hepatic cells showed apoptosis. Moreover, vasculitis and endotheliosis affected some portal areas. The majority of portal areas showed intense portal fibrosis and inflammation, as revealed by mononuclear cells infiltration and contain proliferative bile ductules. In *T*. *fischeri* (100 mg extract/kg) treated group, moderate microscopic changes represented by diffuse acute cells welling and mild portal fibrosis were encountered (Figure 11C). The portal vasculatures were dilated and showed lymphocytosis and scattered minute microsteatosis in individualized hepatic cells. The remaining hepatic parenchyma was apparently normal. Pretreatment with 200 mg/kg of the extract exerted a better improvement in the structural changes of the liver where only portal inflammation was observed (Figure 11D). The effect of the known liver protectant silymarin was similar to the 200 mg/kg dose of the extract (Figure 11E).

To sum up, pronounced antioxidant and hepatoprotective effects were observed in different experimental models in vitro and in vivo in *C. elegans* and in rats. These results are in agreement with those reported for extracts containing cinchonains and phenylpropanoid-substituted cinchonains from *Trichilia catigua* and *Parabarium huaitingii* [14,15].

## 3. Materials and Methods

### 3.1. Plant Material and Extraction

The bark of *Turraea fischeri* was collected from Lupaga Site in Shinyanga, Tanzania. A sample is stored at IPMB, Heidelberg University under the accession number P7335. The dried and ground stembark (100 g) was defatted with n-hexane and then extracted with methanol (100%) at ambient temperature (3 × 500 mL). The filtrates were combined and evaporated under vacuum at 40 °C until dryness. The obtained residue was lyophilized yielding fine dried powder (21 g).

### 3.2. HPLC-PDA-MS/MS

The LC system was Thermo Finnigan (Thermo Electron Corporation, Waltham, MA, USA). The reversed-phase column (Zorbax Eclipse XDB-C18, rapid resolution, 4.6 × 150 mm, 3.5 µm, Agilent, Santa Clara, CA, USA) was used. The mobile phase was water and acetonitrile (ACN) (0.1% formic acid each) and gradient was employed from 5 % to 30% CAN in 60 min with flow rate 1 mL/min with a 1:1 split before the ESI source. Autosampler surveyor ThermoQuest (Thermo Electron Corporation, Waltham, MA, USA) was used to inject the sample; the process was controlled by Xcalibur software (Xcalibur^TM^ 2.0.7, Thermo Scientific, Waltham, MA, USA) [23]. LCQ-Duo ion trap mass spectrometer (ThermoQuest Corporation, Austin, TX, USA) with an ESI source (ThermoQuest) was used and operated in the negative mode as described before [22]. Full scan mode with a mass range of 50–2000 *m*/*z* was employed to detect the ions.

### 3.3. Antioxidant Activities

In vitro antioxidant properties were determined according to DPPH radical scavenging activity and ferric reducing antioxidant power (FRAP) assays, as well as total phenolic content; they were done as reported before [24]. In vivo antioxidant potential was determined in *Caenorhabditis elegans*. The *C. elegans* strains [Wild type (N2), TJ375 [hsp-16.2::GFP (gpls1)] and TJ356] were obtained from the Caenorhabditis Genetic Center (CGC). The worms were maintained at 20 °C on nematode growth medium (NGM), and fed with living *E. coli* OP50. The cultures were synchronized by treating gravid adults with sodium hypochlorite. In M9 buffer, the eggs were hatched and obtained larvae were moved into S-media seeded with living *E. coli* OP50 (D.O600 = 1.0). For in vivo assays, in the survival rate, initially, the worms (N2) were incubated with three different concentrations (25, 50, 100 µg) for 48 h, and then juglone (80 µM) was added for another 24 h. The worms then were counted and the results were expressed as a mean of a percentage of live nematodes. Again, to determine the intracellular ROS concentration, the worms were incubated with three different concentrations of the extract for 48 h. The worms were then incubated in M9 buffer contained 20 μM H2DCF-DA at 20 °C for 1 h. Using fluorescence microscope (Keyence, BZ-9000, Osaka, Japan), the intracellular ROS concentration was measured using fluorescence intensity and then quantified by ImageJ 1.48 software (National Institutes of Health, Bethesda, MD, USA). Quantification of *Phsp*-16.2::GFP expression was studied in TJ375 strains while subcellular DAF-16::GFP localization was studied in the transgenic strain TJ356. All of the analyses were carried out according to our protocols described in more details [11,12,25].

### 3.4. Hepatoprotective Experiments

#### 3.4.1. Animals

Thirty healthy male Wistar rats weighing 180–200 g were utilized in this study and were obtained from the animal house of the Faculty of Veterinary medicine, University of Zagazig, Zagazig, Egypt. The animals were kept in clean polypropylene cages under standard conditions (temperature: 25 °C; 12/12 h light dark cycle; relative humidity: about 50%). A seven-day acclimatization period was allowed before the experiment and the rats were allowed free access to food and tap water. All of the treatments were in accordance with the Guide for the Care and Use of Laboratory Animals of National Academy of Sciences, USA (2011) [26]. Approval (number P5-6-2016) was granted by the Animal Ethics Committee of the Faculty of Pharmacy, Zagazig University prior to commencement of the study.

Rats were randomly assigned into five experimental groups. Each group contained equal numbers of male rats (i.e., *n* = 6). Group (A) represents control group and was given a 1 mL single oral dose of vehicle, while group (B) received 800 mg/kg D-galactosamine (D-GaIN) dissolved in normal saline by intraperitoneal injection. Group (C) received a single oral dose of *T. fischeri* extract (100 mg/kg b.w.). Group (D) received single oral dose (200 mg/kg b.w.) of *T. fischeri* extract. Group (E) represents a positive control, which received a single oral dose of silymarin (100 mg/kg b.w.), a known liver protecting natural product. One hour later, the animals of groups (C, D, and E) received 800 mg/kg D-galactosamine (D-GaIN) dissolved in normal saline by intraperitoneal injection. The extract and silymarin were suspended in gum acacia (10 mg/mL saline *w*/*v*). Identical control groups were utilized in conjunction with our previously published article [23].

#### 3.4.2. Blood and Tissue Sampling

24 h after D-GalN injection, rats were euthanized following anesthesia using diethylether (Fisher Scientific UK Ltd., Loughborough, UK) and 3 mL of venous blood were collected from each animal from the retro-orbital plexus in a plain tube. All of the blood samples were centrifuged (3000× *g*, 4 °C, and 15 min) and the serum was separated and used to measure the levels of liver enzymes alanine aminotransferase (ALT), aspartate aminotransferase (AST), and gamma-glutamyltransferase (GGT), and total bilirubin.

For histopathology and oxidative stress measurement, a liver specimen of 1 cm length × 0.5 cm width × 0.5 µm thicknesses was excised from each liver and washed off with cold saline. It was divided into two parts. One part was immediately flash frozen in liquid nitrogen and kept at −80 °C for measurements of oxidative stress parameters, reduced glutathione and lipid peroxidation product, and malondialdehyde (GSH and MDA, respectively). The other part was processed embedded in paraffin and used for histopathological examination.

#### 3.4.3. Biochemical Determinations

The serum levels of ALT, AST, GGT, and total bilirubin in the rats were measured using a colorimetric kit according to the instructions of the company (Diamond Co., Cairo, Egypt and Spectrum Co., Cairo, Egypt). One gram liver tissues were homogenized. Then lipid peroxidation product, malondialdehyde (MDA) and reduced glutathione (GSH) content were measured in tissue homogenate photometrically (spectrophotometer, Jenway®, Stone, UK) according to Ohkawa et al. [27] and Beutler et al. [28], respectively.

#### 3.4.4. Histology Studies

Serial sections of 4–5 μm thickness were cut from each tissue block, mounted on slides, and kept at room temperature. Then they were dewaxed in xylene, hydrated using graded ethanol, and stained with hematoxylin and eosin (H&E) (Abcam, Cambridge, MA, USA) to assess hepatic architecture and structural changes. All of the sections were examined with an EVOS XL core microscope (Thermo Fisher Scientific, Waltham, MA, USA) at ×100 and ×400 magnifications. The sections were photographed with a digital camera (Canon, Tokyo, Japan).

### 3.5. Statistical Analysis

The values are presented as the means ± SEM. Significant differences between all of the groups were assessed by one-way analysis of variance using the Tukey post hoc-test test and unpaired *t* test. A value of *p* < 0.05 was considered significant. A computer-based curve fitting program (GraphPad Prism 5, Inc., La Jolla, CA, USA) was used to perform statistical analysis.

## 4. Conclusions

In this study, we characterized the phytoconstituents of a methanol bark extract of *T*. *fischeri.* Altogether, 20 compounds were tentatively identified belonging to cinchonains and phenylpropanoid-substituted catechin. *T*. *fischeri* extract demonstrated substantial antioxidant and hepatoprotective properties. *T. fischeri* could be further studied to isolate its biologically-active phytoconstituents and to study their mechanism of action in more detail as this plant shows promising liver-protecting activities.

## Figures and Tables

**Table 1:** Tentative identification of secondary metabolites of a methanol extract of *T. fischeri* bark using HPLC-PDA-MS/MS in the negative ion mode.

No.	*t*_R_	[M − H]^−^	MS/MS Fragments	Tentatively Identified Compounds
**2**	2.00	353	179, 191	Chlorogenic acid
**1**	1.65	447	315, 153	Protocatechuic acid pentosyl-hexoside
**15**	33.41	451	299, 341	Cinchonain-I *
**8**	25.24	613	299, 341, 451, 595	Cinchonain-I hexoside *
**9**	26.27	613	299, 341, 451, 595	Cinchonain-I hexoside *
**10**	27.69	613	299, 341, 451, 595	Cinchonain-I hexoside *
**6**	22.39	759	299, 323, 433, 451, 613, 649	Cinchonain-I rhamnosyl-hexoside *
**7**	23.00	759	299, 323, 433, 451, 613, 649	Cinchonain-I rhamnosyl-hexoside *
**11**	27.18	759	299, 323, 433, 451, 613, 649	Cinchonain-I rhamnosyl-hexoside *
**12**	28.27	759	299, 323, 433, 451, 613, 649	Cinchonain-I rhamnosyl-hexoside *
**13**	30.11	759	299, 323, 433, 451, 613, 649	Cinchonain-I rhamnosyl-hexoside *
**17**	36.1	759	299, 323, 433, 451, 613, 649	Cinchonain-I rhamnosyl-hexoside *
**16**	34.76	775	305, 393, 461, 503, 613, 665	Bis-dihydroxyphenylpropanoid-substituted catechin hexoside **
**20**	47.74	775	305, 393, 461, 503, 613, 665	Bis-dihydroxyphenylpropanoid-substituted catechin hexoside **
**5**	12.24	793	341, 299, 451, 503, 613, 639	Cinchonain-I syringyl-hexoside *
**14**	31.45	921	305, 461, 485, 595, 613, 811	Bis-dihydroxyphenylpropanoid-substituted catechin rhamnosyl-hexoside **
**18**	38.77	921	305, 461, 485, 595, 613, 811	Bis-dihydroxyphenylpropanoid-substituted catechin rhamnosyl-hexoside **
**19**	44.64	921	305, 461, 485, 595, 613, 811	Bis-dihydroxyphenylpropanoid-substituted catechin rhamnosyl-hexoside **
**3**	8.54	939	289, 613, 759	Bis-dihydroxyphenylpropanoid-substituted catechin syringyl-rhamnoside **
**4**	9.72	939	289, 433, 613, 759	Bis-dihydroxyphenylpropanoid-substituted catechin syringyl-rhamnoside **

* indicates that the stereochemistry of the compounds is not resolved; this compound could be cinchonain-Ia, Ib, Ic, or Id. ** indicates that the stereochemistry of the compounds is not resolved. The compounds are arranged based on their molecular weights. Compounds are numbered in accordance to Figure 1.

**Table 2:** Antioxidant properties of a methanol extract from *T. fischeri* bark.

Sample	Bark Extract	AscorbicAcid
DPPH (EC_50_, µg/mL)	5.12	3.31
FRAP (mM FeSO_4_/mg extract)	18.32	22
