# Correction: Sobeh et al. HPLC-PDA-MS/MS Characterization of Bioactive Secondary Metabolites from *Turraea fischeri* Bark Extract and Its Antioxidant and Hepatoprotective Activities In Vivo. *Molecules* 2017, *22*, 2089

**DOI:** 10.3390/molecules29143319

**Published:** 2024-07-15

**Authors:** Mansour Sobeh, Mona F. Mahmoud, Omar M. Sabry, Rasha Adel, Malak Dmirieh, Assem M. El-Shazly, Michael Wink

**Affiliations:** 1 Institute of Pharmacy and Molecular Biotechnology, Heidelberg University, Im Neuenheimer Feld 364, 69120 Heidelberg, Germany; Dmirieh@stud.uni-heidelberg.de; 2 Department of Pharmacology and Toxicology, Faculty of Pharmacy, Zagazig University, Zagazig 44519, Egypt; mona_pharmacology@yahoo.com; 3 Department of Pharmacognosy, College of Pharmacy, Cairo University, Cairo 11562, Egypt; omar.sabry@pharma.cu.edu.eg; 4 Department of Pharmacognosy, Faculty of Pharmacy, Zagazig University, Zagazig 44519, Egypt; rashaadelelfeqy@gmail.com (R.A.); assemels2002@yahoo.co.uk (A.M.E.-S.)

The authors wish to emphasize that the experiments were simultaneously conducted with several plant species, and identical control groups were utilized for both articles (*Phytomedicine* (2017), doi:10.1016/j.phymed.2017.07.003 [1] (reference [23] in the original publication), and *Molecules* (2017), doi:10.3390/molecules22122089 [2]). This decision was made in accordance with the ethical guidelines aimed at minimizing animal usage. By maintaining consistent control groups across multiple studies, we were able to reduce the number of animals needed for experimentation while ensuring robust and reliable results. Despite using the same control data, it is important to note that the results presented in each article represent distinct analyses with unique interpretations and conclusions. We have taken steps to ensure clarity and transparency in reporting our findings by providing detailed methodologies and statistical analyses in each publication.

Both articles share the same approval number, 6-2016, where “P” denotes the plant number. Specifically, “P2” signifies plant number 2, which corresponds to *Ximenia americana*, while “P5” designates plant number 5, referring to *Turraea fischeri*.

In the original publication, we did not mention that the control groups were utilized in conjunction with our previously published article (*Phytomedicine* (2017), doi:10.1016/j.phymed.2017.07.003) [1]. The authors apologize for not disclosing this similarity in the original submission.

In the original publication [2], there was a mistake in the legend for Figure 11. The correct legend appears below.

**Figure 11:** Representative photomicrographs of cross sections from six rat livers (staining with hematoxylin and eosin, 400×); (**A**) Liver of healthy rats with normal hepatocytes arranged in hepatic cords radiating from the central vein with normal portal area, central vein and normal ducts; (**B**) Liver of D-galactosamine treated rats showing focal hepatic necrosis infiltrated by mononuclear cells (arrow head) with microsteatosis of the adjacent hepatocytes (arrows); (**C**) Liver of extract (100 mg/kg) treated rats with minute fibrous strands in portal area and interlobular tissue with reversible degenerative changes mainly vacuolar degeneration in the hepatic cells (arrows); (**D**) Liver of extract (200 mg/kg) treated rats showing portal inflammation (arrows) but no steatosisornecrosis; and, (**E**) Liver of silymarin extract (100 mg/kg) treated rats showing partial improvement but some monocellular infiltration. Photomicrographs from control, D-galactosamine and silymarin groups were published before in [23], where the experiments were carried out in parallel.

## Text Correction

3.4.1. Animals

Rats were randomly assigned into five experimental groups. Each group contained equal numbers of male rats (i.e., *n* = 6). Group (A) represents control group and was given a 1 mL single oral dose of vehicle, while group (B) received 800 mg/kg D-galactosamine (D-GaIN) dissolved in normal saline by intraperitoneal injection. Group (C) received a single oral dose of *T. fischeri* extract (100 mg/kg b.w.). Group (D) received single oral dose (200 mg/kg b.w.) of *T. fischeri* extract. Group (E) represents a positive control, which received a single oral dose of silymarin (100 mg/kg b.w.), a known liver protecting natural product. One hour later, the animals of groups (C, D, and E) received 800 mg/kg D-galactosamine (D-GaIN) dissolved in normal saline by intraperitoneal injection. The extract and silymarin were suspended in gum acacia (10 mg/mL saline *w*/*v*). Identical control groups were utilized in conjunction with our previously published article [23].

The authors state that the scientific conclusions are unaffected. This correction was approved by the Academic Editor. The original publication has also been updated.

## References

[1] Sobeh M., Mahmoud M.F., Abdelfattah M.A.O., El-Beshbishy H.A., El-Shazly A.M., Wink M. (2017). Hepatoprotective and Hypoglycemic Effects of a Tannin Rich Extract from Ximenia Americana Var. Caffra Root. Phytomedicine.

[2] Sobeh M., Mahmoud M.F., Sabry O.M., Adel R., Dmirieh M., El-Shazly A.M., Wink M. (2017). HPLC-PDA-MS/MS Characterization of Bioactive Secondary Metabolites from *Turraea fischeri* Bark Extract and Its Antioxidant and Hepatoprotective Activities In Vivo. Molecules.

